# Socio-economic determinants of household food security and women’s dietary diversity in rural Bangladesh: a cross-sectional study

**DOI:** 10.1186/s41043-015-0022-0

**Published:** 2015-07-10

**Authors:** Helen Harris-Fry, Kishwar Azad, Abdul Kuddus, Sanjit Shaha, Badrun Nahar, Munir Hossen, Leila Younes, Anthony Costello, Edward Fottrell

**Affiliations:** 1 UCL Institute for Global Health, 30 Guilford Street, London, WC1N 1EH UK; 2 Perinatal Care Project, Diabetic Association of Bangladesh, 122 Kazi Nazrul Islam Avenue, Dhaka, 1000 Bangladesh

**Keywords:** Household food security, Nutrition assessment, Determinants, MAHFP, Dietary diversity, Bangladesh

## Abstract

**Background:**

There has been limited decline in undernutrition rates in South Asia compared with the rest of Asia and one reason for this may be low levels of household food security. However, the evidence base on the determinants of household food security is limited. To develop policies intended to improve household food security, improved knowledge of the determinants of household food security is required.

**Methods:**

Household data were collected in 2011 from a randomly selected sample of 2,809 women of reproductive age. The sample was drawn from nine unions in three districts of rural Bangladesh. Multinomial logistic regression was conducted to measure the relationship between selected determinants of household food security and months of adequate household food provisioning, and a linear regression to measure the association between the same determinants and women’s dietary diversity score.

**Results:**

The analyses found that land ownership, adjusted relative risk ratio (RRR) 0.28 (CI 0.18, 0.42); relative wealth (middle tertile 0.49 (0.29, 0.84) and top tertile 0.18 (0.10, 0.33)); women’s literacy 0.64 (0.46, 0.90); access to media 0.49 (0.33, 0.72); and women’s freedom to access the market 0.56 (0.36, 0.85) all significantly reduced the risk of food insecurity. Larger households increased the risk of food insecurity, adjusted RRR 1.46 (CI 1.02, 2.09). Households with vegetable gardens 0.20 (0.11, 0.31), rich households 0.46 (0.24, 0.68) and literate women 0.37 (0.20, 0.54) were significantly more likely to have better dietary diversity scores.

**Conclusion:**

Household food insecurity remains a key public health problem in Bangladesh, with households suffering food shortages for an average of one quarter of the year. Simple survey and analytical methods are able to identify numerous interlinked factors associated with household food security, but wealth and literacy were the only two determinants associated with both improved food security and dietary diversity. We cannot conclude whether improvements in all determinants are necessarily needed to improve household food security, but new and existing policies that relate to these determinants should be designed and monitored with the knowledge that they could substantially influence the food security and nutritional status of the population.

## Background

Undernutrition is in decline globally [1], yet, in Bangladesh, chronic undernutrition remains high with 24 % of women of reproductive age undernourished [2]. An underlying cause of undernutrition is household food insecurity [1]; Bangladesh has the lowest availability of calories per capita in South Asia [3]. With a projected increase in the incidence of erratic weather events such as flooding and drought, climate change poses particular risks to future domestic agricultural productivity and subsistence-level food production in Bangladesh [4]. To compound this problem, our understanding of the determinants of household food security remains largely theoretical and any policies aimed at improving household food insecurity will be developed from a limited evidence base.

This paper aims to improve our understanding of household food security in Bangladesh. Focussing on rural areas in the districts of Bogra, Faridpur and Moulavibazar we describe the status and socioeconomic determinants of household food security, and the relationship between these determinants, adequacy in household food provisioning and dietary diversity among women of reproductive age.

## Methods

**Fig. 1:**
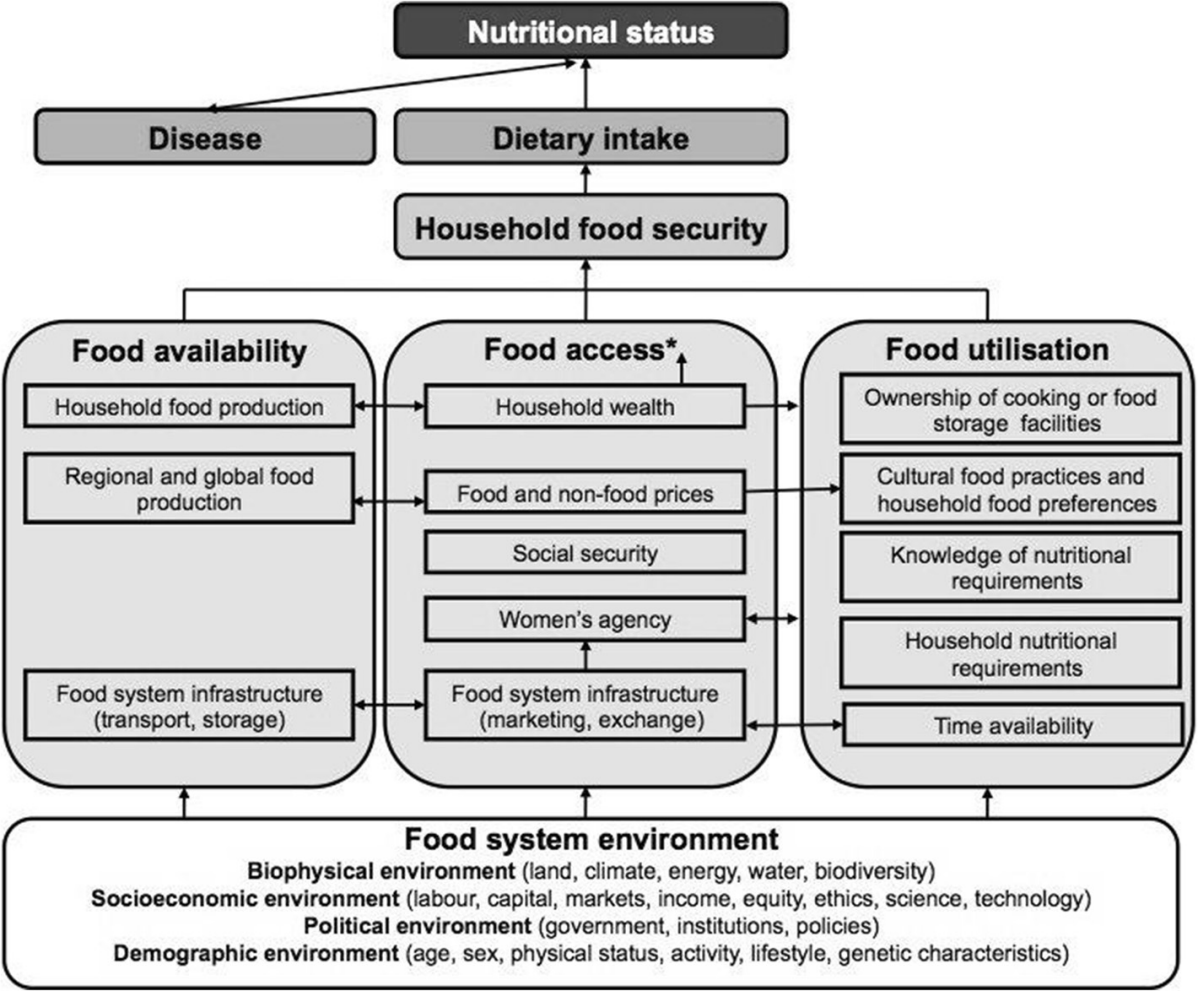
Hypothetical determinants of household food security and nutritional status. *Household wealth is hypothesised to interact with all other food access determinants. For presentation purposes, the framework does not illustrate how household food security and nutritional status can reciprocally impact upon the determinants; this will need to be explored if attempting to determine causality

Our theoretical framework of the determinants of household food security in Fig. 1 is based on the UNICEF undernutrition framework, which illustrates how food availability, access and utilisation are the three ‘pillars’ of food security [1], and Pinstrup-Andersen’s framework of food security linkages [5]. This framework categorises determinants and enables us to explore variables likely to affect household food security. One determinant is the complex concept of agency, for which we adopt Sen’s definition as “what a person is free to do and achieve in pursuit of whatever goals or values he or she regards as important” [6].

### Study setting and population

The research was conducted in nine unions of Bogra, Faridpur and Moulavibazar districts in Bangladesh, covering a sample of 2,809 women of reproductive age (15–49 years). Bogra is located to the north of Dhaka in a plain and has fertile soils. The northerly study sites in this district are widely dispersed and human resource capacity is relatively limited. Faridpur is south of Dhaka and features many large rivers that make it susceptible to flooding and make some areas difficult to access. Moulavibazar, in eastern Bangladesh, is a hilly district, posing challenges for travel. It is characterised by tea garden estates with tea garden workers who are mostly poor and landless.

The sites were selected based on criteria for cluster randomised controlled trials that were conducted by the Diabetic Association of Bangladesh (BADAS) and University College London and are described elsewhere [7–9]. Briefly, districts were purposively sampled according to their proximity to BADAS offices and, within each district, six unions were randomly allocated to the control or intervention arm, resulting in nine intervention and nine control clusters. The sample described in this paper is women from the control arm where there was no intervention other than low intensity health system strengthening initiatives, and is likely to be more representative of the rural Bangladeshi population.

### Sample size and sampling

The random sample of women included in this study represents the control arm sample of a baseline survey taken as part of a formative evaluation of an intervention to improve women’s and reproductive health. The sample size of the control arm therefore relates to the intervention sample size and was powered for a quasi-experimental study to be reported elsewhere. Stratified random sampling was used to select the sample from lists of all households with women of reproductive age and children under five.

### Data collection

From October to December 2011, 36 trained, local data collectors conducted a cross-sectional survey of women using a piloted structured questionnaire. Data were collected on women’s socio-economic status, dietary diversity, knowledge on healthy diets, and women’s autonomy and decision-making ability.

Respondents were interviewed in their homes and approximately 10 % of questions from 10 % of the questionnaires were crosschecked by supervisors who re-visited the households. Questionnaires were checked for completeness in the district BADAS offices before being sent for data entry at BADAS headquarters in Dhaka. The data were also reviewed at BADAS headquarters by surveillance and data managers and inconsistencies were reported to the district offices for correction or verification. Data were entered into a Microsoft Access database and further checked.

### Analysis

Indicators and proxy measures of food security determinants were categorised under food availability, access and utilisation (Fig. 1), and are listed in Table 1.

**Table 1:** Indicators and proxy measures of food security determinants

Food security determinant	Indicator or proxy measure	Survey question
		• Response option
**Food availability**		
Household food production	Ownership of land	Does your household own any land (other than the homestead land)?
		• Yes; No; Don’t know
	Ownership of livestock	Does your household own any livestock, farm animals or poultry?
		• Yes; No; Don’t know
	Use of vegetable gardens	Does your household grow its own fruits and vegetables in a homestead garden or plot?
		• Yes; No; Don’t know
Regional and global food production	Not available	Not available
Food system infrastructure	Not available	Not available
**Food access**		
Household wealth	Principal Component Analysis	Which of these do you presently have in your household?
		• Electricity; fan; mobile phone; non-mobile phone; fridge; almirah/wardrobe; table; chair/bench; cot/bed; mattress; sewing machine; watch/clock; generator; bicycle; motorcycle/scooter/tempo; animal-drawn cart; car/truck/bus/microbus; boat; rickshaw/van
		What is the main material of the floor in the house where the woman lives? (Record your observation)
		• Earth/sand; wood planks; palm/bamboo; parquet or polished wood; ceramic tiles; cement; carpet; other; don’t know.
		What is the main material of the roof in the house where the woman lives? (Record your observation)
		• No roof; thatch/palm leaf; bamboo; wood planks; cardboard; tin; wood; ceramic tiles; cement; stone with lime/cement; roofing shingles; other; don’t know.
		What is the main material of the exterior walls in the house where the woman lives? (Record your observation)
		• No walls; cane/palm/trunks/straw; dirt; bamboo/bamboo with mud; stone/stone with mud; plywood; cardboard; tin; cement; stone with lime/cement; bricks; wood planks/shingles; other; don’t know.
		Does your household own any homestead? *If no, probe*: Does your household own homestead in any other places?
		• Yes; No; Don’t know
Food and non-food prices	Not available	Not available
Social security	Not available	Not available
Women’s agency	Freedom for women to always or sometimes access the market by herself	Are you allowed to go to the market/ shops without the company of another adult?
		• Always; sometimes; never allowed to go without company; never allowed to go even with company
	Involvement in decision-making relating to daily household expenditures (by herself or with her husband).	Who has the greatest say in the decision regarding how household money is spent for daily necessities, such as food?
		• Woman; woman and her husband jointly; husband; mother-in-law; father-in-law; mother; father; sister-in-law; brother-in-law; other family members; other; don’t know.
Food system infrastructure	Not available	Not available
Ownership of cooking or food storage facilities	Not available	Not available
**Food utilisation**		
Knowledge of nutritional requirements	Knowledge of malnutrition prevention (number of methods named)	What can women do to stay well nourished? What else? Do not prompt. (Multiple answer)
		• Eat adequate amount of nourishing/vitamin rich food every day; eat plenty of vegetables; eat eggs/milk/dairy products; prevent childhood (and early) marriage; prevent adolescent pregnancy; eat more food during pregnancy; adequate rest during pregnancy; prevent frequent pregnancy/space births by more than 2 ½ years; increase food quantity during lactation; maintain hygiene and cleanliness/look after one’s health; other (specify); don’t know.
	Women’s literacy	Can you read this passage for me please?
		(*Interviewer to decide the ability level)*
		• Easily; with difficulty; cannot read.
	Access to media (ownership of radio or television) Taken from asset score, which is partially used in the PCA.	Which of these do you presently have in your household?
		• Radio/tape recorder; television with cable; television without cable.
Household size	Not available	Not available
Larger household	Number of women of reproductive age in the household	How many women aged 15–49 live in your household?
		• Number of women
Intra-household food distribution and food control	Not available	Not available
Cultural practices and individual practices	Not available	Not available

Indicators of household food security included a measure of household food shortages, Months of Adequate Food Provisioning (MAHFP), and an individual-level Women’s Dietary Diversity Score (WDDS). MAHFP is the number of months per year that households reported no food shortages and was calculated according to Food and Nutrition Technical Assistance Project (FANTA) guidelines [10]. WDDS uses the following nine food group indicators: starchy staples, legumes and nuts, dairy, organ meat, eggs, flesh meat and fish, dark green leafy vegetables, other vitamin A-rich vegetables and fruits, and other fruits and vegetables [11]. Respondents reported whether or not they had eaten each food group over the last 24 h.

Socio-demographic variables measured were quality of wall, roof and floor materials in the home, ownership of homestead land and ownership of 22 assets such as electricity, bicycle or sewing machine. We used a Principal Component Analysis (PCA) to derive relative wealth groups, divided into tertiles. Some variables such as ownership of land, livestock and some assets were not included in the PCA because they were hypothesised (in Fig. 1) to be separate determinants of household food security that are independent of wealth.

MAHFP data were not normally distributed, with heaping at 0 and 12 months. Based on this, we deemed a logical categorisation of low, moderate and high food insecurity to be <9, 9–11, and 12 MAHFP respectively. Another study also used this categorisation, based on the assumption that extreme values are meaningful [12]. We used multinomial logistic regression to assess crude associations between the food security determinant variables and MAHFP. Crude associations with WDDS were assessed using linear regression. On the basis that socio-demographic characteristics and food security determinants may confound associations being tested, we controlled for these factors in multivariate regression models adjusting for age, religion and pregnancy status and all other measured food security determinants.

To test for collinearity between variables, we calculated the binary association between variables using an adjusted Wald test and the multivariate variance inflation factors (VIFs).

Analyses were conducted in Stata/IC 12.1 and we used the svyset function with weighting to account for the stratified cluster survey sampling used. Results with a p value of < = 0.05 were considered statistically significant.

### Ethics

Ethical approval was obtained from the ethics committees of BADAS, Dhaka, Great Ormond Street Hospital and Institute of Child Health (GOSH-ICH), London. Women who chose to participate in the study gave verbal consent and were free to stop the interview at any time.

## Results

We obtained responses from 93 % of the target sample. Reasons for not successfully interviewing the women were because the respondents were not at home, the woman had divorced, or the respondent had died. We were unable to check for response bias due to the lack of data on non-responders. 6.6 % (n 189) of women were excluded from the WDDS analysis because they had celebrated or feasted within the previous 24 h. Reporting of a feast day was not associated with any household characteristics or variables used in the analysis, except households with more than one woman of reproductive age were less likely to report a feast (*χ*^2^ = 5.25, *p =* 0.04). We expect that this is a spurious finding because we cannot identify any rationale for bigger households feasting less, since wealth is not associated. Nevertheless, interpretation of results must be done with the usual caution applied to sample surveys with incomplete response.

### Study population characteristics

The study population characteristics are summarised in Table 2. All respondents were women of reproductive age, with a mean age of 30.8 years (SD 8.0; range 15, 49). 90 % of respondents were Muslim; the remaining 10 % were Hindu. Approximately two thirds of respondents were literate.

**Table 2:** Summary of respondent characteristics

Characteristic	Bogra	Faridpur	Moulavibazar	Total % (n)
**Total**	*n =* 818	*n =* 1,163	*n =* 828	100 *n =* 2,809
**Age (years)**	*n =* 818	*n =* 1,163	*n =* 827	*n =* 2,808
Mean	30.6 (SD = 8.1)	30.2 (SD = 7.7)	32.8 (SD = 8.2)	30.8 (SD = 8.0)
≤19	7.5 (61)	4.0 (47)	3.0 (25)	5.3 (133)
20–24	18.0 (147)	21.7 (252)	14.1 (117)	18.5 (516)
25–29	24.1 (197)	26.6 (309)	21.6 (179)	24.5 (685)
30–34	18.3 (150)	19.2 (223)	22.1 (183)	19.5 (556)
≥35	32.2 (263)	28.6 (332)	39.1 (324)	32.4 (918)
**Religion**	*n =* 818	*n =* 1,163	*n =* 828	*n =* 2,809
Islam	93.9 (768)	93.1 (1083)	78.4 (649)	90.3 (2500)
Hindu	6.1 (50)	6.9 (80)	21.6 (179)	9.7 (309)
**Pregnancy status**	*n =* 818	*n =* 1,163	*n =* 828	*n =* 2,809
Pregnant	5.1 (42)	5.7 (66)	5.0 (41)	5.3 (149)
Not pregnant	94.9 (776)	94.3 (1097)	95.1 (787)	94.7 (2660)
**PCA wealth score** ^a^	*n =* 814	*n =* 1,160	*n =* 824	*n =* 2,798
Lowest tertile	26.3 (214)	34.2 (397)	39.0 (321)	31.9 (932)
Middle tertile	39.7 (323)	28.5 (331)	26.2 (216)	32.8 (870)
Top tertile	34.0 (277)	37.2 (432)	34.8 (287)	35.4 (996)
Mean number of assets owned	7.0 (2.6)	7.0 (3.0)	6.7 (SD = 3.0)	6.9 (SD = 2.8)
**Educational status**	*n =* 818	*n =* 1,163	*n =* 828	*n =* 2,809
None or less than 1 year	51.3 (420)	56.8 (660)	55.2 (457)	54.1 (1,537)
Primary (any level)	23.5 (192)	18.5 (215)	21.3 (176)	21.2 (583)
Secondary and higher	25.2 (206)	24.8 (288)	23.6 (195)	24.7 (689)
**Literacy**	*n =* 818	*n =* 1,163	*n =* 828	*n =* 2,809
Cannot read	37.5 (307)	33.6 (391)	36.2 (300)	35.5 (998)
Can read (easily or with difficulty)	62.5 (511)	66.4 (772)	63.8 (528)	64.5 (1,811)

^a^0.04 % missing data for PCA wealth score

### Household food security

**Fig. 2:**
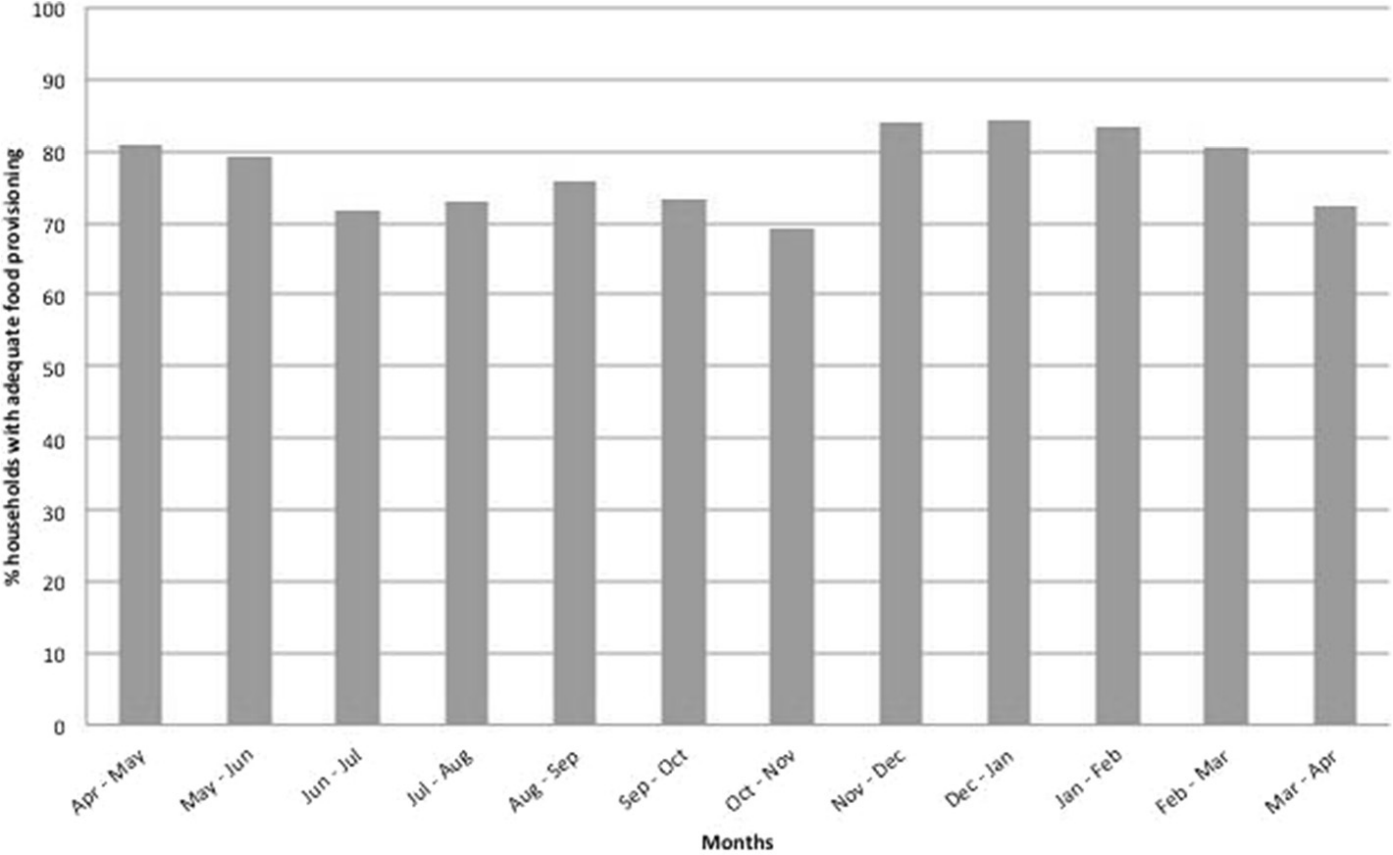
Percentage of respondents with adequate household food provisioning between 2010 and 2011

The percentage of respondents reporting adequate household food provisioning in 2010–11 is displayed by Gregorian months in Fig. 2. The month with the highest proportion (almost one third) of households facing food shortages was Kartik (October to November); in Agrahaiyan (November to December) food shortages fell sharply to 16 %. Over half of respondents reported no food shortages over the year. Of those that did, respondents faced shortages for an average of half of the year and 54.7, 19.1, and 26.2 % of households had 12, 9–11 and <9 months of adequate food provisioning respectively.

**Fig. 3:**
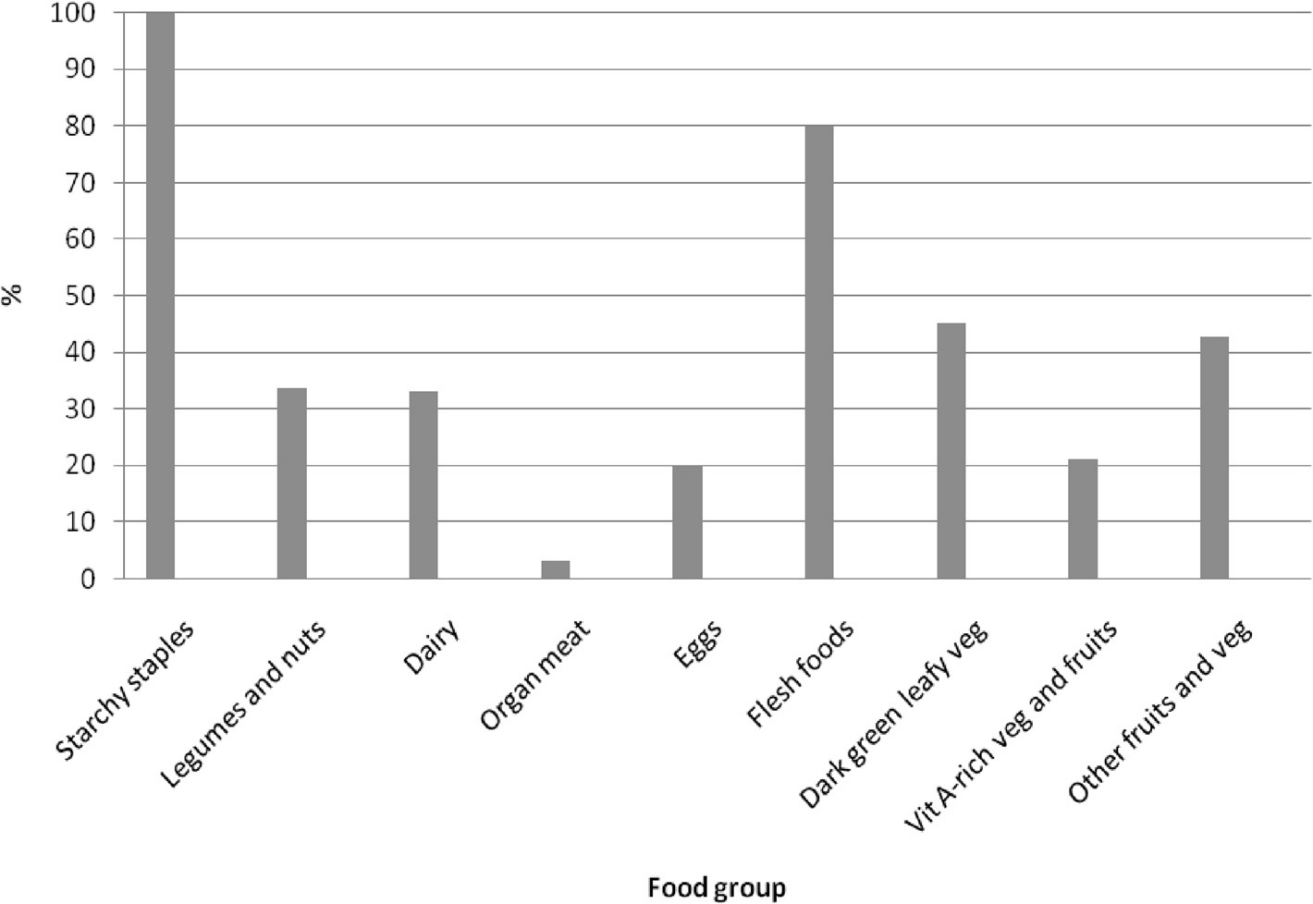
Respondents’ consumption of food groups over 24 h

Respondents had a mean WDDS of 3.8 (range 1, 9). Consumption of food groups by all women is shown in Fig. 3. There was no significant difference in WDDS or consumption of food groups depending on pregnancy status of women (results not shown).

### Food availability determinants

The determinants of food availability are listed in Table 1. Five percent of respondents reported that they owned land. Of those who owned land, respondents owned an average of 0.7 acres (range 0.05, 22.1). Eighty percent of respondents reported owning livestock; farm animals, or poultry and they owned on average 9 animals (range 1, 106). Just over half of respondents reported use of a homestead garden.

### Food access determinants

Respondents reported owning an average of 7 (range 0, 16) out of 22 assets, such as electricity or a table.

Women’s freedom to shop alone was limited; almost half of respondents were never allowed to shop without company, and 6 % were never allowed to shop, with or without the company of another adult. Sixty three percent of respondents reported that they (alone or with their husband) were the main decision-makers for purchasing daily necessities such as food.

### Food utilisation determinants

Respondents could name an average of 2 (range 0, 9) ways for women to stay well-nourished. The commonest methods listed were to eat plenty of vegetables; to eat eggs, milk or dairy products; and to eat an adequate amount of nourishing, vitamin-rich food every day. Most respondents (86 %) thought that women should eat more than usual during pregnancy, and 5 % thought women should eat less. Over one third of respondents reported ownership of a radio or a television.

### Analysis of the determinants of MAHFP and WDDS

Testing for collinearity between determinants, we found an expected positive and significant association between relative wealth (PCA score) and land ownership (F_1,6_ = 132.71, *p =* <0.000), ownership of livestock (F_1,6_ = 21.37, *p =* 0.004) and access to media (F_1,6_ = 474.80, *p =* <0.000). However, VIFs were sufficiently low, ranging between 1.03 and 2.16, indicating that the inclusion of the separate determinants in the model is statistically valid.

Table 3 shows results from crude and adjusted multinomial logistic regression analysis of the associations between measures of food security determinants and MAHFP. Determinants of food availability, access and utilisation were associated with food insecurity. Wealth and land ownership were the strongest protective factors against food insecurity; both factors reduced the risk of low food security by more than 70 % and 80 % respectively. Literacy and access to media also significantly reduced the risk of food insecurity, with the likelihood of low food security being up to a third lower amongst the literate and 50 % lower where households had access to media. The ownership of livestock and the women’s freedom to go to the market alone were protective against severe food insecurity but there was no evidence of an association with mild food insecurity. Having more than one woman of reproductive age living in the same household was associated with an increased risk of severe food insecurity in the adjusted analysis and age was associated with a slight increase in all categories of food insecurity.

**Table 3:** Associations between possible food security determinants and months of adequate household food provisioning

Possible determinants of MAHFP	Total	MAHFP	Crude^a^				Adjusted^b^			
			Moderate food security		Low food security		Moderate food security		Low food security	
	Weighted % (n)	mean (95 %CI)	RRR (95 % CI)	p value	RRR (95 % CI)	p value	RRR (95 % CI)	p value	RRR (95 % CI)	p value
**Total**	(2808)	9.3 (range 0, 12)								
**Respondent characteristics**										
**Age**	(2808)		1.03 (1.01, 1.04)	0.007	1.04 (1.03, 1.04)	0.000	1.02 (1.01, 1.04)	0.005	1.04 (1.03, 1.05)	0.000
**Religion**										
Islam^c^	90.3 (2499)	9.3 (8.2, 10.4)								
Hindu	9.7 (309)	8.9 (7.6, 10.2)	1.16 (0.44, 3.04)	0.715	1.16 (0.57, 2.37)	0.632	1.47 (0.60, 3.65)	0.335	1.31 (0.55, 3.12)	0.481
**Pregnancy status**										
Not pregnant^c^	94.7 (2659)	9.3 (8.2, 10.3)	0.64 (0.32, 0.74)	0.176	1.01 (0.74, 1.39)	0.932	0.76 (0.43, 1.35)	0.289	1.12 (0.78, 1.61)	0.464
Pregnant	5.3 (149)	9.2 (7.6, 10.7)								
**Food availability determinants**										
**Ownership of land**										
Does not own land^c^	53.7 (1534)	8.0 (6.2, 8.9)								
Owns land	46.3 (1271)	10.7 (10.1, 11.3)	0.35 (0.28, 0.44)	0.000	0.19 (0.14, 0.28)	0.000	0.47 (0.39, 0.57)	0.000	0.28 (0.18, 0.42)	0.000
**Ownership of livestock**										
Does not own livestock^c^	19.9 (598)	7.8 (6.3, 7.3)								
Owns livestock	80.1 (2210)	9.6 (8.6, 10.6)	0.87 (0.58, 1.32)	0.453	0.46 (0.37, 0.57)	0.000	1.13 (0.71, 1.81)	0.543	0.65 (0.51, 0.82)	0.004
**Use of vegetable gardens**										
Do not use gardens^c^	48.3 (1293)	9.1 (7.8, 10.4)								
Use gardens	51.8 (1515)	9.4 (8.3, 10.5)	0.54 (0.28, 1.04)	0.062	0.79 (0.41, 1.54)	0.427	0.65 (0.40, 1.07)	0.081	1.05 (0.57, 1.94)	0.853
**Food access determinants**										
**Wealth (PCA)**										
Lowest tertile^c^	31.9 (931)	7.2 (5.5, 8.8)								
Middle tertile	32.8 (870)	9.5 (8.3, 10.6)	0.61 (0.42, 0.90)	0.020	0.34 (0.22, 0.51)	0.001	0.81 (0.56, 1.19)	0.230	0.49 (0.29, 0.84)	0.018
Top tertile	35.4 (996)	11.0 (10.7, 11.3)	0.20 (0.10, 0.37)	0.001	0.08 (0.05, 0.14)	0.000	0.36 (0.18, 0.70)	0.010	0.18 (0.10, 0.33)	0.000
**Woman’s freedom travel to market alone**										
Never^c^	62.9 (1835)	8.9 (7.8, 10.1)								
Always or sometimes	37.1 (973)	9.8 (8.9, 10.8)	1.19 (0.50, 2.86)	0.639	0.61 (0.39, 0.96)	0.037	1.12 (0.48, 2.61)	0.759	0.56 (0.36, 0.85)	0.015
**Woman’s participation in decision-making relating to daily expenditures**										
No^c^	63.2 (1794)	9.2 (7.9, 10.5)								
Yes	36.8 (1014)	9.4 (8.5, 10.3)	1.42 (0.99, 2.02)	0.053	1.04 (0.58, 1.86)	0.886	1.35 (0.88, 2.08)	0.139	1.07 (0.66, 1.71)	0.751
**Food utilisation determinants**										
**Knowledge of undernutrition prevention methods (number of methods known)**										
0–2^c^	76.2 (2101)	9.2 (7.9, 10.4)								
≥3	23.8 (707)	9.6 (8.9, 10.3)	0.85 (0.39, 1.86)	0.631	0.86 (0.46, 1.58)	0.560	1.10 (0.57, 2.14)	0.728	1.05 (0.58, 1.90)	0.854
**Women’s literacy**										
Cannot read^c^	35.9 (998)	7.9 (6.5, 9.4)								
Can read	64.2 (1810)	10.0 (9.2, 10.9)	0.36 (0.27, 0.49)	0.000	0.27 (0.19, 0.39)	0.000	0.64 (0.44, 0.95)	0.031	0.64 (0.46, 0.90)	0.019
**Access to media**										
No radio or television^c^	63.9 (1815)	8.5 (7.2, 9.8)								
Own radio or television	36.1 (993)	10.7 (10.0, 11.3)	0.34 (0.24, 0.50)	0.000	0.22 (0.15, 0.33)	0.000	0.56 (0.35, 0.90)	0.024	0.49 (0.33, 0.72)	0.004
**Number of women of reproductive age in the household**										
1 woman^c^	76.9 (2115)	9.2 (8.1, 10.3)								
>1 woman	23.1 (693)	9.4 (8.5, 10.4)	0.90 (0.64, 1.28)	0.505	0.95 (0.82, 1.10)	0.427	1.30 (0.82, 2.08)	0.214	1.46 (1.02, 2.09)	0.041

We have a maximum of 0.04 % missing data on background information and food utilization determinants, a maximum of 0.14 % missing data on food availability determinants, and a maximum of 0.43 % missing data on food access determinants

^a^Adjusted for clustering and stratification only

^b^Adjusted for socio-demographic characteristics (age and religion), pregnancy status and the other determinants of food security

^c^Reference group

Table 4 shows results from linear regression analysis of associations between possible food security determinants and WDDS. The adjusted analysis found that use of vegetable gardens, increasing wealth, literacy and increasing household size were significantly associated with increases in dietary diversity scores of between 0.21 and 0.5.

**Table 4:** Associations between possible food security determinants and women’s dietary diversity score

Possible determinants of WDDS	Total	WDDS	Crude^a^		Adjusted^b^	
	% (n)	Mean (95 % CI)	Coeff. (95 % CI)	p value	Coeff. (95 % CI)	p value
**Total**	100 (*n =* 2620)	3.8 (range 1,9)				
**Respondent characteristics**						
**Age**	2620		−0.01 (−0.01, 0.00)	0.036	0.00 (−0.01, 0.01)	0.973
**Religion**						
Islam^c^	90.2 (2328)	3.7 (3.6, 3.9)				
Hindu	9.8 (292)	4.2 (3.7, 4.6)	0.42 (−0.06, 0.90)	0.075	0.32 (0.00, 0.65)	0.052
**Pregnancy status**						
Not pregnant^c^	94.7 (2480)	3.8 (3.7, 3.9)				
Pregnant	5.3 (140)	4.0 (3.6, 4.5)	0.26 (−0.13, 0.65)	0.154	0.21 (−0.07, 0.48)	0.118
**Food availability determinants**						
**Ownership of land**						
**Does not own land** ^***c***^	54.2 (1443)	3.6 (3.5, 3.7)				
Owns land	45.8 (1174)	4.0 (3.9, 4.1)	0.39 (0.30, 0.48)	0.000	0.10 (−0.01, 0.21)	0.070
**Ownership of livestock**						
Do not own livestock^c^	19.6 (552)	3.7 (3.6, 3.9)	0.06 (−0.10, 0.23)	0.369	−0.06 (−0.21, 0.08)	0.327
Own livestock	80.4 (2068)	3.8 (3.7, 3.9)				
**Use of vegetable gardens**						
Do not use gardens^c^	48.3 (1199)	3.6 (3.5, 3.8)				
Use gardens	51.7 (1421)	3.9 (3.8, 4.1)	0.32 (0.19, 0.46)	0.001	0.20 (0.11, 0.31)	0.003
**Food access determinants**						
**Wealth (PCA)**						
Lowest tertile^c^	32.2 (876)	3.4 (3.3, 3.5)				
Middle tertile	32.8 (815)	3.7 (3.5, 3.9)	0.33 (0.11, 0.55)	0.011	0.17 (−0.03, 0.37)	0.083
Top tertile	35.0 (918)	4.2 (4.0, 4.3)	0.85 (0.59, 1.12)	0.000	0.46 (0.24, 0.68)	0.002
**Freedom travel to market alone**						
Never^c^	63.0 (1718)	3.8 (3.6, 4.0)				
Always or sometimes	37.0 (902)	3.8 (3.5, 4.0)	−0.05 (−0.47, 0.36)	0.763	−0.04 (−0.40, 0.33)	0.810
**Women’s involvement in decision-making relating to household food expenditure**						
No	63.2 (1668)	3.8 (3.7, 3.9)				
Yes	36.8 (952)	3.8 (3.6, 4.0)	0.02 (−0.20, 0.23)	0.849	0.03 (−0.16, 0.21)	0.731
**Food utilisation determinants**						
**Knowledge of undernutrition prevention methods (number of methods known)**						
0-2	76.3 (1963)	3.7 (3.6, 3.9)				
≥3	23.7 (657)	4.0 (3.7, 4.4)	0.30 (−0.09, 0.68)	0.112	0.14 (−0.16, 0.45)	0.296
**Women’s literacy**						
Cannot read^c^	36.2 (940)	3.4 (3.3, 3.5)				
Can read	63.8 (1680)	4.0 (3.9, 4.1)	0.59 (0.46, 0.72)	0.000	0.37 (0.20, 0.54)	0.002
**Access to media**						
Do not own radio or television	64.1 (1698)	3.6 (3.5, 3.7)				
Own radio or television	35.9 (922)	4.2 (3.9, 4.4)	0.58 (0.27, 0.88)	0.004	0.24 (−0.01, 0.50)	0.056
**Number of women of reproductive age in the household**						
1 woman^c^	77.4 (1985)	3.7 (3.6, 3.8)				
>1 woman	22.6 (635)	4.0 (3.8, 4.2)	0.33 (0.22, 0.43)	0.000	0.17 (0.05, 0.29)	0.012

We have a maximum of 6.73 % missing data on background information and food utilization determinants, a maximum of 6.84 % missing data on food availability determinants, and a maximum of 7.12 % missing data on food access determinants

^a^Adjusted for clustering and stratification only

^b^Adjusted for socio-demographic characteristics (age and religion), pregnancy status and the other determinants of food security

^c^Reference group

## Discussion and conclusion

Our data show that populations in Bogra, Faridpur and Moulavibazar had sufficient food in the household (MAHFP) for only 9.3 (range 0, 12) months over a year, between 2010 and 2011. The highest prevalence of food shortages corresponds with the main harvest; after this there was a sharp fall in food shortages. Women in the household also reported consumption of an average of 3.8 (range 1, 9) food groups over one day. Another study found a comparable WDDS of 4.3 in Bangladesh [13], but what the WDDS means in public health terms is unclear because there are no cut-off points; new guidelines, developed after our survey, propose five out of ten different food groups as ‘adequate’ [14].

After adjusting for confounders, the food security determinants associated with a reduced risk of mild household food insecurity were household food production (measured by ownership of land), household wealth (highest tertile), and knowledge of nutritional requirements (women’s literacy and access to media). The determinants associated with reduced risk of high food insecurity were the same as above, as well as ownership of livestock, both middle and highest wealth tertiles, women’s agency (freedom to travel to the market), and household size (fewer women of reproductive age in the household). Determinants associated with increased WDDS were household food production (use of vegetable gardens), wealth (top tercile), ownership of cooking or food storage facilities (refrigerator ownership), knowledge of nutritional requirements (women’s literacy) and household size.

### Strengths and limitations of the research

The large sample, geographical representation of the sample and simple metrics used provide a useful snapshot household food security and its proximate determinants, and this study contributes to the scarce literature on measures of household food security [12, 15]. The very high response rate is also a notable strength of our study, although being cross-sectional we were unable to assess any bias that from non-responders. The cross-sectional design also prevents an analysis of temporal associations and causality. Since nutritional outcomes could reciprocally affect food security determinants, reverse causality is possible. Prospective monitoring of food security and its determinants is required to elucidate the direction of causation. Also, the measures used do not quantify all hypothetical determinants; this limits the study in the comprehensiveness of the analysis and possibility of missing confounders.

MAHFP is limited in its representation of ‘household food security’ because it captures the respondents’ perceptions of whether they had enough food; MAHFP might only measure calorie, but not micronutrient, security. Therefore, WDDS was used as a measure of household access to a micronutrient-rich diet. Although WDDS is a measure of individual dietary intake, intra-household food distribution in Bangladesh tends to be biased against women, so WDDS is likely to be a conservative estimate of household nutritional security [16, 17]. Future studies could use a Household Dietary Diversity Score, [18] or the Household Food Insecurity Access Scale [19].

### Determinants of household food availability

The strong, significant association between ownership of land and reduced risk of food insecurity is consistent with publications from other parts of the world [20] but is also of particular concern in Bangladesh, given the population growth and decreasing land available per capita. This indicates a continued importance of the promotion of equitable land tenure laws and employment schemes for the landless poor.

The use of vegetable gardens is interesting because it had no association with MAHFP but did show a weak association with WDDS. It is possible that gardens only improve household food intake in seasons when crop yields are high, food prices are lower, and households are already food secure. The latter association with WDDS is consistent with findings that a vegetable garden programme in Bangladesh improved macronutrient and micronutrient consumption [21].

### Determinants of household food access

Respondents in the highest wealth tertile were significantly less likely to experience food shortages. An international review supports this [22], as does a study that found Bangladeshi cash-for-work programmes increased food consumption and nutritional status [23]. We also found an association between wealth and WDDS, reflecting findings from a comparative study from Kenya, the Philippines and Bangladesh [24].

Given control over food purchasing, women tend to prioritise expenditure of household resources on food [25, 26]. Our results showed risks of food insecurity were lower if women could access the market, but no effect was found for involvement in budgetary decisions—maybe because the measures used are crude representations of complex concepts of decision-making and empowerment.

### Determinants of household food utilisation

We hypothesised that access to media and literacy would improve food security by increasing access to and comprehension of health-promoting mass media campaigns. Although access to media and literacy were associated with a reduced risk of food shortages, and literacy was associated with higher dietary diversity, women’s nutritional knowledge was not associated with MAHFP or WDDS. This is surprising since we expect literacy and access to media to have an impact through its effect upon women’s nutrition knowledge [27] and it would appear that literacy and media access affect food security through alternative pathways.

### Policy implications

This research provides a starting point for further quantification of the largely assumed, hypothetical determinants of household food security. To be comprehensive, exploration of other determinants in the framework is required. Research to validate the use of MAHFP would add to early discussions on its appropriateness as a new measure of household food security [28, 29].

Our cross-sectional research identifies numerous possible food security determinants relating to land tenure, use of vegetable gardens, income generation and women’s empowerment. Although we cannot conclude whether improvements in all determinants are required to improve household food security, we have shown the multi-dimensionality of the determinants of household food security. Therefore, policies relating to these determinants, irrespective of their primary objectives, should be designed with the knowledge that they could influence the food security and nutritional status of the population.
